# Post-stroke depression: Prevalence, associated factors and impact on quality of life among outpatients in a Nigerian hospital

**DOI:** 10.4102/sajpsychiatry.v24i0.1058

**Published:** 2018-03-22

**Authors:** Osunwale D. Oni, Andrew T. Olagunju, Victor O. Olisah, Olatunji F. Aina, Francis I. Ojini

**Affiliations:** 1 Department of Psychiatry, Lagos University Teaching Hospital, Nigeria; 2 Department of Psychiatry, College of Medicine, University of Lagos, Nigeria; 3 Department of Psychiatry, Ahmadu Bello University Teaching Hospital, Nigeria; 4 Department of Medicine, University of Lagos, Nigeria

## Abstract

**Objectives:**

To investigate the prevalence of post-stroke depression (PSD), its associated factors and impact on quality of life (QoL) among outpatients in a Nigerian hospital.

**Methods:**

This cross-sectional study was carried out among 140 adults made up of 70 stroke survivors and matched controls with stable hypertension. Participants were administered questionnaires to profile their socio-demographic and clinical characteristics. Subsequently, they were assessed with the modified mini-mental state examination (MMSE), modified Rankin Scale (mRS), schedule for clinical assessment in neuropsychiatry (SCAN) and World Health Organization Quality of Life-BREF (WHOQoL-BREF).

**Results:**

The mean ages (± s.d.) of stroke survivors and controls were 57.43 (± 9.67) years and 57.33 (± 9.33) years, respectively. Majority of stroke survivors (*n* = 55 [78.6%]) had infarctive stroke, and 37 (52.9%) had right hemispheric lesion. Sixteen (22.9%) stroke survivors had PSD, with moderate to severe depression (F32.1) being the most prevalent, while none of the controls was clinically depressed. PSD correlated positively with monthly health bill above 10 000 naira ($61), significant post-stroke disability and poorer scores on all QoL domains (*p* < 0.05).

**Conclusion:**

Depression was 20-fold prevalent in stroke survivors compared to controls with stable hypertension, and sevenfold the life-time prevalence reported among adult general population in Nigeria. Furthermore, increased health care bills per month, significant post-stroke disability and poorer QoL indicated survivors more likely to have depression. Findings in this study support the need to pay closer attention to psychosocial needs of stroke survivors to improve well-being. Future longitudinal study on psychosocial burden of stroke is warranted.

## Introduction

Stroke is a leading cause of death and neurological disability that imposes heavy burden on families.^1^ In 2013, there were 25.7 million stroke survivors, 6.5 million stroke deaths and 10.3 million first-ever strokes worldwide.^2^ The developing countries are worst affected as the World Health Organization (WHO) estimate suggested a sevenfold increase in disability-adjusted life years attributable to stroke in low- and middle-income regions compared to high-income regions.^3^ Recent Nigerian studies have shown the leading contributions of stroke to neurological admissions posing significant burden on caregivers because of prolonged hospitalisation, disability, cost implications of treatment and lost productivity.^4,5^

Diseases of the vascular system like stroke have been linked with significant contribution to psychiatric co-morbidities. Mental health problems post stroke can result from direct brain damage or because of maladaptive reactions to stroke sequelae. In any of the scenarios, interaction between personality makeup and adverse life situations often influences how survivors adjust to disability and in turn impact the rehabilitation process.^6^ The common mental health morbidities post stroke include anxiety, depression, personality changes and psychotic disorders among others.^7,8^ Depressive reactions are frequent among stroke survivors, particularly as they deal with the abrupt and ‘novel’ experience of living with stroke that is often frightening and ill understood.^9^ More often, survivors are unable to evaluate their situation objectively, which may lead to projection of blame outwardly or towards self.^9^ In the acute phase of stroke, depression has been attributed to survivor’s cognitive interpretation of physical disabilities, enforced dependency on others and uncertainty about recovery as well as prognosis.^6^ On the contrary, long-term risk factors for post-stroke depression (PSD) include job status, financial insecurity and permanent loss of independence among others.^6^

Earlier reviews showed PSD to be the commonest mental disorder in survivors, with up to 25.5% prevalence rate reported in previous local studies.^10,11,12^ Additionally, studies have shown significant associations between emotional disorders like depression and poorer quality of life (QoL) in stroke survivors.^13,14^ Such comorbidity of stroke with depression has been described as the ‘double burden’ of stroke because both depression and stroke are leading contributors to total years lost to disability based on the global burden of diseases report.^15^ In fact, PSD is consistently linked with poor psychosocial outcome and increased mortality in survivors.^13,14,15,16^ Unfortunately, PSD is often poorly diagnosed and under reported, in part because cognitive problems after stroke can confound the symptoms of depression and make diagnosis difficult.^17,18^ Care providers may also consider depressive symptoms a natural consequence of sudden physical disability after stroke that will eventually resolve without treatment.^17^

Despite increased interest in the psychosocial aspects of chronic medical conditions in developing countries, not much is known about PSD in sub-Saharan Africa. There is also a dearth of literature on the impacts of PSD on QoL, which may hamper the formulation of broad policy to ensure holistic care of stroke survivors. To address current gaps, our study is aimed at investigating the prevalence of PSD, its associated clinico-demographic factors and impacts on QoL among outpatients in a Nigerian hospital. We posited that the occurrence of PSD would be influenced by clinico-demographic factors and impact negatively on QoL.

## Methods

### Study design and setting

This case-control cross-sectional study was carried out among 70 adults with stroke and equal number of matched controls with controlled hypertension at the Lagos University Teaching Hospital (LUTH), Lagos, Nigeria. Data collection was carried out over a period of 5 months from October 2013 to February 2014. The study sample size was calculated using the public domain statistical software Java-Stat with power set at 80%.^19^ Recruitment of participants was performed using non-probability convenience sampling of every eligible participant who met the study criteria until estimated sample size was completed. Of a total of 196 participants made up of stroke survivors and controls approached for the study, 19 declined consent and 37 did not meet the study criteria.

Study participants were stroke survivors (aged 18 years and above) with either first-ever or recurrent strokes. They were recruited from follow-up neurology and physiotherapy clinics after obtaining informed consent. Stroke diagnosis was ascertained with neuroimaging (computerised tomography [CT] or magnetic resonance imaging [MRI]) investigations and clinical judgement of the managing neurologists as documented in participants’ case folders. Excluded from the study were stroke survivors with background history of depression prior to indexed stroke (either first-ever or recurrent strokes), stroke survivors with current mental health disorder(s) besides depression, those with severe cognitive deficits (mini-mental state score < 16), severe communication problems (i.e. severe aphasia), stroke survivors on antihypertensives (like methyldopa and reserpine) that could precipitate depression and those with chronic debilitating medical conditions besides hypertension and uncomplicated diabetes. Information on physical health, past mental health and medication use was obtained from case records of participants. Data obtained during clinical interviews were also documented.

Controls were stable individuals with controlled hypertension matched for age and sex of the recruited stroke survivors. They must have had normal blood pressures at least 3 months prior to data collections (information regarding this was obtained from case folders). Recruitment of controls was from the general outpatient department clinic (GOPD) of LUTH. Exclusion criteria for controls included history of other mental disorders, chronic debilitating physical co-morbidities and use of antihypertensive medications like reserpine and methyldopa.

### Instruments and procedure

The study instruments included socio-demographic and clinical questionnaire (SCQ), modified mini-mental state examination (MMSE), schedule for clinical assessment in neuropsychiatry (SCAN), modified Rankin Scale (mRS) and World Health Organization Quality of Life-BREF (WHOQoL-BREF).^20,21,22,23^

The SCQ was designed by the authors and administered to obtain socio-demographic and clinical data. Other relevant clinical information was obtained from case notes. Stroke subtypes and lateralisation were determined from CT scan or MRI results. In the absence of radiological scans, stroke subtype was categorised as indeterminate, and lateralisation was based on clinical assessment performed by the managing neurologist. The MMSE assesses cognitive function and was originally developed by Folstein but has since been modified for local use.^20^ The MMSE was administered to participants to measure cognitive impairments and rule out severe communication problems. We then carried out semi-structured clinical interview with SCAN to evaluate, classify and measure psychopathology and behaviour.^21^ In SCAN interviews we compare the subjective experience of participants elicited by the interviewer against a glossary of descriptive clinical phenomenon. The elicited phenomena are then used to generate clinical diagnoses based on International Classification of Diseases, tenth edition (ICD-10), criteria using data imputed imputes into the SCAN algorithm software.^21,24^ The screening section of SCAN was used to check the possibility of a depressive disorder and rule out other mental disorders. Study participants were thereafter interviewed with sections with probes to diagnose depression.^24^ The mRS was then used to measure the degree of disability or dependence in daily activities in study participants.^22^ Health-related QoL was measured with the 26-item self-administered WHOQoL-BREF questionnaire.^23^ The controls after being matched with the study sample were administered SCQ and SCAN to diagnose depression and other current mental illnesses.

The authors were trained in the use of SCAN by certified tutors prior to data collection. The SCAN, MMSE, mRS and WHOQoL-BREF has been validated and used extensively in Nigeria.^11,20,25,26,27,28,29^

### Data analyses

All data collected were coded and entered into the computer for analysis. Data were analysed using the Statistical Package for Social Sciences (SPSS) for windows Version 17.0 software.^30^ Mean (± SD), frequencies and percentages were used to describe data. Independent *t*-test was used to compare continuous variables. Chi-square test analysed significance between categorical variables, and Fischer’s exact test was used when the criteria for chi-square were not met. A confidence interval of 95% was used which allows for 5% sampling error at significance of ≤ 0.05.

## Ethical consideration

Ethical approval was obtained from the Health Research and Ethics Committee (HREC) of Lagos University Teaching Hospital (LUTH) before commencement of the study. Informed consent was given by all participants following explanation of study purpose, procedure and expected duration of involvement. Other issues like confidentiality and voluntariness regarding participation were also explained. Those with clinically significant depression were counselled and referred appropriately.

## Results

### Socio-demographic and clinical characteristics of participants

A total of 140 respondents took part in the study. This consisted of 70 stroke survivors (study sample) and 70 stable patients with hypertension (control sample). The study also had 38 (54.3%) male respondents and 32 (45.7%) female participants each in both samples. Among stroke survivors, 91.4% had radiological investigations in the form of CT or MRI scans. Six (8.5%) participants without radiological scans had their stroke subtype categorised indeterminate, and lateralisation was determined via clinical assessment. Also 78.6% and 12.9% had infarctive and haemorrhagic strokes, respectively. In the same vain, 52.9%, 41.4% and 5.7% of participants had right hemispheric, left hemispheric and brain stem-posterior column lesions, respectively. All participants in the study sample were right handed.

Table 1 showed socio-demographic characteristics of the study participants and controls. There was no significant difference in the mean ages of stroke survivors (57.43 [± 9.67] years) and controls (57.33 [± 9.33] years). Controls were not significantly different from stroke survivors in all socio-demographic parameters reported (*p* < 0.0.5).

**TABLE 1:** Socio-demographic characteristics of participants.

Parameter	Study group Mean (± s.d.)		Controls Mean (± s.d.)		TOS
	*n*	%	*n*	%	
**Mean age by gender**					
Total (*n* = 70, 100.0%)	57.43	9.67	57.33	9.33	*t* = 0.062 *p* = 0.95
Male (*n* = 38, 54.3%)	59.41	10.29	57.29	9.39	*t* = 0.944 *p* = 0.35
Female (*n* = 32, 45.7%)	54.94	8.34	57.38	9.40	*t* = −1.089 *p* = 0.28
**Age class (years)**					
31–40	2	2.9	2	2.9	χ^2^ = 0.056
41–50	16	22.9	16	22.9	*df* = 4
51–60	26	37.1	25	35.7	*p* = 0.99
61–70	21	30.0	22	31.4	-
71–80	5	7.1	5	7.1	-

**Total**	**70 (100.0)**	**-**	**70 (100)**	**-**	**-**

χ^2^ = chi square; s.d., standard deviation; *t*, independent *t* test; df, degree of freedom; TOS, test of significance.

*p* = level of significance < 0.05

### Prevalence and severity of depression among study samples

Table 2 presented results on prevalence of depression. The prevalence of depression in stroke survivors (PSD) was 22.9% (*n* = 16) and none in the control group were diagnosed with depression (*p* < 0.001). Among the stroke survivors with depression, a majority 10 (62.5%) had moderate depressive illness (F 32.1) with 3 (18.8%) and 1 (6.3%) having mild (F 32.0) and severe (F 32.2) depressive episodes, respectively. Also, 2 (12.4%) of stroke survivors with PSD had dysthymia (F 34.1).

**TABLE 2:** Prevalence and severity of depression with International Classification of Diseases, tenth edition codes among samples.

Parameter	Study		Control		TOS
	*n*	%	*n*	%	
**Depression**					
Yes	16	22.9	0	0.0	χ^2^* = 18.065
No	54	77.1	70	100	***p* < 0.001**
**Severity of depression (ICD-10 codes)**					
Mild depressive episode (F32.0)	3	18.8	0	0.0	-
Moderate depressive episode (F32.1)	10	62.5	0	0.0	-
Severe depressive episode (F32.2) without psychoses	1	6.3	0	0.0	-
Dysthymia (F34.1)	2	12.4	0	0.0	-

TOS, test of significance; χ^2^* = Fischer’s exact; ICD-10, International Classification of Diseases, 10th edition.

*p* = level of significance < 0.05, *p* in bold = statistically significant

### Comparison of socio-demographic characteristics of participants with and without post-stroke depression

Considering all the socio-demographic characteristics, stroke survivors with PSD were not significantly different from those without PSD, except with regard to monthly health bills. Participants with PSD were over-represented among those that spent more than 10 000 naira ($61) per month (*p* < 0.001) on health care bills. Despite absence of significant difference, participants with PSD tend towards having lower mean age (54.06 [± 9.94] years), being female (9 [20.1%]) and unemployed (10 [35.7%]) (see Table 3).

**TABLE 3:** Comparison of socio-demographic characteristics between study participants with and without post-stroke depression.

Parameter	Study sample *N* = 70	Post-stroke depression		TOS
		Yes (*n* = 16)	No (*n* = 54)	
**Mean age**	57.43 (± 9.67)	54.06 (± 9.94)	58.43 (± 9.45)	*t* = −1.603 *p* = 0.11
**Sex**				
Male	38 (54.3)	7(10.4)	31 (89.6)	χ^2^ = 0.93
Female	32 (45.7)	9(20.1)	23 (79.9)	*df* = 1 *p* = 0.34
**Marital status**				
Married	56 (80.0)	13 (23.2)	43 (76.8)	χ^2^ = 0.02*
Unmarried	14 (20.0)	3 (21.4)	11 (78.6)	*df* = 1 *p* = 0.89
**Educational level**				
Secondary or less	41 (58.6)	7 (17.1)	34 (82.9)	χ^2^ = 1.08
Tertiary	29 (41.4)	9 (31.0)	20 (69.0)	*df* = 1 *p* = 0.17
**Employment**				
Employed	22 (31.4)	3 (13.6)	19 (86.4)	χ^2^ = 3.982*
Retired	20 (28.6)	3 (15.0)	17 (75.0)	*p* = 0.15
Unemployed	28 (40.0)	10 (35.7)	18 (64.3)	
**Monthly health bill (₦)**				
< 10 000	36 (54.3)	2 (5.6)	34 (94.4)	χ^2^ = 12.583
> 10 000	34 (45.7)	14 (41.2)	20 (58.8)	*df* = 1
***p* < 0.001**

s.d., standard deviation; *t*, independent *t*-test; *df*, difference of freedom; χ^2^*, Fischer’s exact; χ^2^ = chi square; Naira (one dollar ~^ **₦** 165 based on 2013 exchange rate).

*p* = level of significance < 0.05; *p* in bold, statistically significant

### Comparison of clinical factors among participants with and without post-stroke depression

Higher proportions of stroke survivors with PSD had infarctive stroke (14 [25.5%]) and left hemispheric stroke (9 [31.0%]). More stroke survivors with PSD were interviewed less than a year after current stroke (10 [35.7%]). All stroke survivors with PSD had significant disability (16 [100.0%]), ranging from slight to severe post-stroke disability on the mRS. The difference in severity of disability between stroke survivors with and without PSD was statistically significant (*p* = 0.03) (see Table 4).

**TABLE 4:** Comparison of clinical characteristics between study participants with and without post-stroke depression.

Parameter	Study group *N* = 70 (%)	Post-stroke depression		TOS
		Yes (*n* = 16)	No (*n* = 54)	
**Stroke subtype**				
Indeterminate	6 (8.5)	1 (16.7)	5 (83.3)	χ^2^* = 0.759
Infarctive	55 (78.6)	14 (25.5)	41 (74.5)	*df = 2*
Haemorrhagic	9 (12.9)	1 (11.1)	8 (88.9)	*p* = 0.78
**Stroke duration (years)**				
< 1	28 (40.0)	10 (35.7)	18 (64.3)	χ^2^* = 4.726
1–2	9 (12.9)	2 (22.2)	7 (77.8)	*df = 2*
> 2	33 (47.1)	4 (12.1)	29 (87.9)	*p* = 0.09
**Stroke lateralisation**				
Rt hemispheric	37 (52.9)	7 (18.9)	30 (81.1)	χ^2^* = *1.937*
Lt hemispheric	29 (41.4)	9 (31.0)	20 (69.0)	*df = 2*
Brain stem/post lobe	4 (5.7)	0 (0.0)	4 (100.0)	*p = 0.60*
**Co-morbidities**				
No	3 (4.3)	0 (0.0)	3 (100.0)	χ^2^* = *2.396*
Hypertension	54 (77.1)	11 (20.4)	43 (79.6)	*df = 2*
Hypertension + diabetes	13 (18.6)	5 (38.5)	8 (61.5)	*p = 0.31*
**Sexual dysfunction**				
Yes	16 (22.9)	3 (18.8)	13 (81.2)	χ^2^ = *1.98*
No	54 (77.1)	13 (23.2)	41 (76.8)	*df = 1* *p = 0.75*
**Sensory impairment**				
Yes	60 (85.7)	13 (21.7)	47 (78.3)	χ^2^* = 0.338
No	10 (14.3)	3 (30.0)	7 (70.0)	*df = 1* *p* = 0.56
**Modified Rankin score**				
No significant disability	13 (18.5)	0 ( 0.0)	13 (100.0)	χ^2^* = 4.730
Significant disability	57 (81.5)	16 (28.1)	41 (71.9)	***p* = 0.03**

χ^2^, chi square; *df*, degree of freedom; χ^2^*, Fischer’s exact; CT, computerised tomographic scan; MRI, magnetic resonance imaging; TOS, test of significance.

*p* = level of significance < 0.05; *p* in bold, statistically significant

### Comparison of quality of life scores among participants with and without post-stroke depression

Findings on QoL are presented in Table 5. Across all domains of QoL, stroke survivors with PSD had significantly lower QoL (*p* < 0.001). The mean (± s.d.) scores of stroke survivors with PSD across the spheres of QoL were overall health (51.25 [± 12.59]), health satisfaction (40.00 [± 12.65]), physical health domain (41.19 [± 9.83]), psychological health domain (41.75 [± 12.64]), social health domain (50.75 [± 8.17]) and environmental health domain (47.12 [± 10.97]).

**TABLE 5:** Comparison of World Health Organization Quality of Life mean scores in study participants with and without post-stroke depression.

WHOQoL domains	Post-stroke depression		TOS
	Yes (mean ± s.d.)	No (mean ± s.d.)	
Overall health	51.25 (± 12.59)	69.19 (± 13.59)	*t* = −5.01 *p* < 0.001
Health satisfaction domain	40.00 (± 12.65)	60.48 (± 17.01)	*t* = −4.65 *p* < 0.001
Physical health domain	41.19 (± 9.83)	59.98 (± 11.80)	*t* = −6.10 *p* < 0.001
Psychological health domain	41.75 (± 12.64)	58.93 (± 11.53)	*t* = −5.55 *p* < 0.001
Social health domain	50.75 (± 8.17)	61.23 (± 12.98)	*t* = −2.15 *p* < 0.001
Environmental health domain	47.12 (± 10.97)	53.52 (± 9.52)	*t* = −2.49 ***p* < 0.001**

s.d., standard deviation; *t*, independent *t*-test; TOS, test of significance.

*p* = level of significance < 0.05; *p* in bold, statistically significant

## Discussion

Our study investigated the prevalence, correlates and impact of PSD on QoL among an outpatient population of stroke survivors in comparison to controls with stable hypertension. Several important lessons which can bridge the existing gap in knowledge, policy formulation and holistic care of stroke survivors were brought to fore. Firstly, prevalence rate of depressive illnesses was significantly higher in stroke survivors compared to control and the life-time prevalence rate reported in Nigerian adult population.^31^ Secondly, both health care spending and severity of post-stroke disability were identifiable correlates of PSD. Thirdly, poorer QoL scores across all spheres were found among stroke survivors with PSD in comparison to those without. These findings are particularly important in sub-Saharan Africa where the care of stroke is mainly focused on controlling blood pressure to prevent recurrent stroke and physiotherapeutic intervention to limit disability, while paying less attention to psychosocial sequelae of stroke.

### Prevalence of post-stroke depression among participants

The prevalence of PSD in this study is 22.9% and represents many folds higher the prevalence of depression previously documented in adult general population in Nigeria and study control with stable hypertension.^31^ The prevalence of PSD in this study is also higher than findings in other populations with chronic medical illnesses.^27,32,33^ Overall, the prevalence rate in our study falls within the range of prevalence rates (19.2% – 30.0%) reported in earlier studies in Nigeria.^11,12,34^ However, higher prevalence rates have been reported in other African studies.^35,36^ Plausible reasons for the discrepancies between our work and other cited studies could be methodological. For instance, some of the studies with higher prevalence rates^35,36^ assessed depression using screening rating scales rather than the structured interviews used in this study.

In terms of severity, moderately severe depression was most prevalent and found in about three-fifths of stroke survivors with PSD in our study. This finding is in agreement with another local study by Ajiboye et al.^11^ where SCAN was also used to diagnose depression and categorise severity. Taken together, this pattern of severity of depressive illness validates previous reports of greater psychological morbidity in chronic physical conditions, thereby underscoring the need for supportive and mental health services in stroke survivors.^37^

### Socio-demographic and clinical correlates of post-stroke depression among participants

Considering the correlates of PSD in our work, stroke survivors with PSD were more likely to pay more for health care and trend towards being unemployed, although this is not statistically significant. In Nigeria, payment for health care is largely out-of-pocket because of poor health insurance coverage. Our findings therefore suggest that stroke survivors with depression were more prone to financial challenges because of unemployment, increased health care bills and virtual lack of insurance coverage.^38^ This may have spiral negative impact on family support systems that shoulder health care cost burden and caregiving. Other studies have found inverse relationships between economic status and occurrence of PSD.^36,39^

Generally, existing literature showed a positive correlation between PSD and presence of significant or severe post-stroke disability in concordance with findings in our study.^10,40,41^ Our study measured severity of post-stroke disability with mRS using scale scoring that ranged from zero to five. Participants were dichotomised to those with no significant disability (based on mRS scores of zero and one) and those with significant disability (based on mRS scores of two to five). This disability rating post stroke by the mRS is based on the ability to carry out certain functions of daily living pre- and post-stroke. Few dissenting findings have been reported. For instance, Zikic et al.^42^ noted that recovery in stroke survivors with depression was less likely compared to those without depression, whereas Sinyor et al.^43^ found that survivors with PSD in the acute stroke phase still remained depressed despite appreciable gains at rehabilitation follow-up. This has often led to the argument that PSD may not be entirely explained as a reaction to disability and reinforced the consideration of the interplay of multi-factorial and bio-psycho-social factors in the aetio-pathogenesis of PSD.^44^

In comparison to findings in previous studies, our study observed that higher proportions of stroke survivors with PSD tend to be females, younger, had left hemispheric lesions and had shorter post-stroke durations at interview, but these findings were not statistically significant. Earlier findings on these aforementioned factors have largely been inconsistent, thereby justifying the need for more studies in order to understand the relationship of these potential risk factors to PSD.^10,11,12,13,14,41,45,46,47,48^

### Relationship between post-stroke depression and quality of life among participants

Stroke being catastrophic in nature has been posited to have significant impact on many aspects of the survivor’s life and well-being. Health-related QoL measures may be more relevant to stroke survivors than measures of disability or impairment. It may also facilitate better description of stroke outcomes and improve communication with patients on a broad range of concepts that include physical health, psychological state, level of independence, personal benefits and their relationship to salient features of the environment where individual resides.^49^ Unlike other chronic disabling illnesses, onset of stroke is sudden, leaving survivors and family ill prepared to deal with the health and psychosocial sequelae.^47^ The impacts of stroke on QoL are multilayered and can affect survivors with mild consequences of stroke and those who have achieved full independence in activities of daily living.^14^ Previous studies have reported variable degrees of impairment across the domains of QoL among stroke survivors in different populations.^14,48,49,50^ Our study found that PSD was associated with statistically significant lower mean scores in all QoL domains. Our finding validates observations of impaired QoL in other earlier studies on one hand and also extends existing literature by reporting the negative interplay of psychiatric morbidity on all domains of QoL post stroke on the other hand.^13,14^

In spite of the importance of our findings, the clinic-based nature and cross-sectional design in this study suggest the need for caution while extrapolating its findings to other stroke survivors and interpretation for causal inferences. Exclusion of certain stroke survivors with severe cognitive and communication deficits, mental disorders and other chronic debilitating physical conditions may also limit the generalisation of our results. Finally, the use of a generic QoL measures rather than stroke-specific QoL measure may affect the accuracy of the study results.

In conclusion, depression was 20-fold prevalent among stroke survivors in comparison to controls with stable hypertension and many folds what has been reported among Nigerian adult general population. Stroke survivors with PSD were more likely to report higher health care costs, significant post-stroke disability and poorer QoL. Hence, screening of stroke survivors for depression using identifiable risk factors as an integral part of their management may have potential benefits. In this regard, training of health care professionals to ensure prompt detection of emotional sequelae of stroke and enhancing their skills to carry out psycho-education to informal caregivers are implied. To enhance prompt detection and targeted intervention, there is need for disease-specific tools to screen psychosocial problems and track outcome among stroke survivors. Further research with robust study design to investigate the complex interplay of PSD on well-being across the stroke recovery trajectory is implied.
